# Daptomycin-Nonsusceptible *Staphylococcus aureus*: The Role of Combination Therapy with Daptomycin and Gentamicin

**DOI:** 10.3390/genes6041256

**Published:** 2015-11-30

**Authors:** Jhih-Hang Jiang, Anton Y. Peleg

**Affiliations:** 1 Infection and Immunity Program, Monash Biomedicine Discovery Institute and Department of Microbiology, Monash University, Clayton, Victoria 3800, Australia; E-Mail: jhih-hang.jiang@monash.edu; 2 Department of Infectious Diseases, The Alfred Hospital and Central Clinical School, Monash University, Melbourne, Victoria 3004, Australia

**Keywords:** *S. aureus*, synergy, time-kill studies, VISA, MRSA, combination therapy

## Abstract

Reduced susceptibility to daptomycin in *Staphylococcus aureus* has now been described, leading to clinical failures. Here we determined the impact of daptomycin and gentamicin combination therapy on bactericidal activity and resistance emergence using daptomycin-susceptible and -resistant isolates with mutations linked to previous daptomycin or vancomycin exposure. Enhanced killing of *S. aureus* was observed when gentamicin was combined with daptomycin, most commonly with daptomycin concentrations below the peak serum free-drug concentrations achieved with standard dosing. Synergy was seen with daptomycin-susceptible isolates and with isolates resistant to vancomycin and daptomycin. Combination therapy also prevented the emergence of resistance. Daptomycin and gentamicin combination therapy may provide the synergy required to prevent emergence of resistance when daptomycin levels are below peak serum concentrations as would be found in deep-seated, complicated infections.

## 1. Introduction

*Staphylococcus aureus* is an opportunistic bacterial pathogen that can cause severe infections in hospital and the community. Due to the emergence of multi-drug resistance in *S. aureus*, treatment now relies on last-line antibiotics, including daptomycin. Daptomycin is a cyclic lipopeptide antibiotic that targets the bacterial membrane for its bactericidal activity, a mechanism similar to host cationic antimicrobial peptides. Unfortunately, therapeutic failures with daptomycin for infections due to *S. aureus* have now been reported [1,2]. The majority of these patients had deep-seated infection, such as endocarditis or osteomyelitis, and increases in minimum inhibitory concentration (MIC) of daptomycin were observed after prolonged therapy [1,2]. It is postulated that the levels of daptomycin may have been sub-therapeutic at the site of infection. In this study, we used the term daptomycin resistance (DAP-R) (daptomycin MIC > 1 μg/mL) instead of daptomycin non-susceptibility.

Studies thus far show that *S. aureus* acquires DAP-R via the accumulation of single nucleotide polymorphisms (SNPs) in genes related to the biogenesis of the cell membrane and cell wall, especially the multi-peptide resistance factor gene *mprF* and the essential two component regulator *walKR* (also known as *yycGF*) [3]. MprF is a lysyl-phosphatidylglycerol (L-PG) synthetase with two functional domains that synthesizes L-PG and facilitates L-PG translocation to the outer leaflet of the cell membrane respectively [4]. SNPs in *mprF* associated with DAP-R cause a gain-in-function and therefore lead to more total L-PG or more L-PG in the outer leaflet of the membrane [5,6,7]. It is hypothesized that this leads to altered membrane charge and electrorepulsion of daptomycin [7]. WalK is the histidine kinase that modulates its cognate transcriptional regulator, WalR, to control cell wall biosynthesis and turnover [8]. Importantly, the emergence of cross-resistance to daptomycin and vancomycin, another last-line antibiotic, is concerning as several vancomycin-intermediate *S. aureus* (VISA) clinical isolates are also reported to be DAP-R even without daptomycin exposure [9,10]. This observation is correlated with a study that recreated SNPs in *walk* and *walR* that were found in VISA clinical isolates, and showed they conferred reduced susceptibility to vancomycin and daptomycin in *S. aureus* [11]. Therefore, novel strategies are required to reduce the chances for deep-seated *S. aureus* infections to develop DAP-R during daptomycin treatment.

The potential benefits of combining daptomycin with aminoglycosides were first observed by Debbia *et al.*, showing synergistic killing of combination therapy against *S. aureus* in time-kill assays [12]. Gentamicin is one of the most active aminoglycosides to treat *S. aureus* infection and has therefore been a common additional agent investigated with daptomycin (Table S1) [13,14,15,16,17,18,19,20,21,22,23,24,25]. A simulated endocardial vegetation (SEV) model has also been used to assess the efficacy of daptomycin plus gentamicin [26]. The combination enhanced bactericidal activity in some SEV studies [16,23] but was indifferent from daptomycin monotherapy in other SEV studies [22,25]. The inconsistent results may be due to differences between bacterial strains and unknown levels of daptomycin penetrating into SEVs. It has previously been shown that ~91%–94% of daptomycin in plasma is bound to proteins, and based on peak daptomycin concentrations (98–133 μg/mL) achieved after standard dosing (6 or 8 mg/kg/day), concentrations of free daptomycin in tissues and bones are reported between 1.6 and 8 μg/mL [27,28,29]. Thus far, less is known about the efficacy of daptomycin-gentamicin combination using daptomycin concentrations likely found in deep-seated infection sites and against isolates that have genetically characterized daptomycin resistance mechanisms [15,17,20,25].

In this study, we investigated the synergy between daptomycin and gentamicin using genetically characterized *S. aureus* DAP-R isolates, as well as DAP-S isolates from patients with deep-seated infections. Two of the DAP-R isolates were only exposed to daptomycin whilst the other two DAP-R isolates were only exposed to vancomycin. Given the majority of infections that fail daptomycin therapy are complicated bacteremia cases with associated deep-seated infection, we chose a clinically relevant range of daptomycin concentrations to assess for enhanced killing with gentamicin.

## 2. Experimental Section

### 2.1. Bacterial Strains

We examined eight *S. aureus* clinical isolates (Table 1), six of which were paired isolates from three patients. Four pre-treatment methicillin-resistant *S. aureus* (MRSA) isolates (A9719, A8819, A8796 and A6224), two VISA strains with DAP-R that emerged after vancomycin therapy (A6226 and A9639), and two DAP-R *S. aureus* isolates that emerged after daptomycin therapy (A9744 and A8817), were included [2,7,10,30]. All isolates underwent whole genome sequencing as described previously [7,10]. The genetic mutations identified by whole-genome analysis between paired DAP-R isolates and DAP-S progenitors in previous studies are shown in Table S2 [7,10].

**Table 1:** *Staphylococcus aureus* isolates used in the present study.

Patient	Strains	Clinical Syndrome	Multi-Locus Sequence Type	MIC (μg/mL)			References
				Daptomycin	Vancomycin	Gentamicin	
1	A6224	Bacteremia	5	0.25	2	0.5	[10]
	A6226			2	4	0.5	
2	A9719	Endocarditis	5	0.25	2	0.5	[7]
	A9744			2	2	1	
3	A8819	Osteomyelitis, septic arthritis	105	0.25	1	0.5	[7]
	A8817			2	1	0.5	
4	A8796	Bacteremia, osteomyelitis	105	0.25	1	0.5	[2,7]
5	A9639	Bacteremia, osteomyelitis	1892	2	4	1	[10,30]
Control	ATCC 29213	−	−	0.25	1	0.5	−

### 2.2. Antibiotic Susceptibility Testing

Daptomycin (Cubist Pharmaceuticals, Lexington, MA, USA) susceptibility testing was performed by broth macrodilution (inoculum, 5 × 10^5^ CFU/mL) using cation-adjusted Mueller-Hinton broth (Beckton Dickinson, Cockeysville, MD, USA) supplemented to contain a final calcium concentration of 50 μg/mL. Susceptibility to vancomycin and gentamicin was performed by agar dilution according to the Clinical and Laboratory Standards Institute (CLSI) [31]. *S. aureus* ATCC 29213 was used as a control.

### 2.3. Time-Kill Analyses

Time-kill studies, as described previously, were performed to assess for enhanced killing [26,32]. An inoculum of 10^6^ CFU/mL of fresh overnight cultures was used. Calcium supplemented (final concentration of 50 μg/mL), cation-adjusted Mueller-Hinton broth was used for all time-kill studies. Bacterial colony counts were performed in duplicate at baseline, 4 h and 24 h after incubation at 35 °C. Two hundred microliters were sampled from the undiluted flask at 24 h resulting in a lower limit of organism detection of 5 CFU/mL. Enhanced killing was defined as a ≥2 log_10_ decrease in CFU/mL between the combination and its most active component after 24 h. At least one of the drugs had to be present in a concentration that did not significantly affect the growth curve of the organism when used alone [32]. For the DAP-S isolates (A6224, A9719, A8819, and A8796), we studied daptomycin concentrations between 0.5 and 6 μg/mL (2× MIC to 20× MIC). For the DAP-R *S. aureus* isolates (A6226, A9639, A9744 and A8817), we studied daptomycin concentrations between 4 μg/mL and 16 μg/mL (2 × MIC to 8× MIC). The concentration of gentamicin in skeletal muscle and subcutaneous tissue varies depending on gentamicin concentration in serum, gender, age, the degree of peripheral arterial disease and pathological state of the tissue [33,34,35]. A range between 0.3 μg/mL and 7 μg/mL gentamicin in skeletal muscle and tissue was reported in the literature after a 3 mg/kg/day dose [33,34,35,36]. To simulate a low gentamicin concentration in deep-seated infection, a gentamicin dose of 0.25 μg/mL or 0.5 μg/mL (0.5× MIC) was used in time-kill analyses.

## 3. Results and Discussion

### 3.1. Synergistic Bactericidal Effects of Gentamicin and Daptomycin at the Concentration Mimicking That in Tissue Compartments

**Figure 1:**
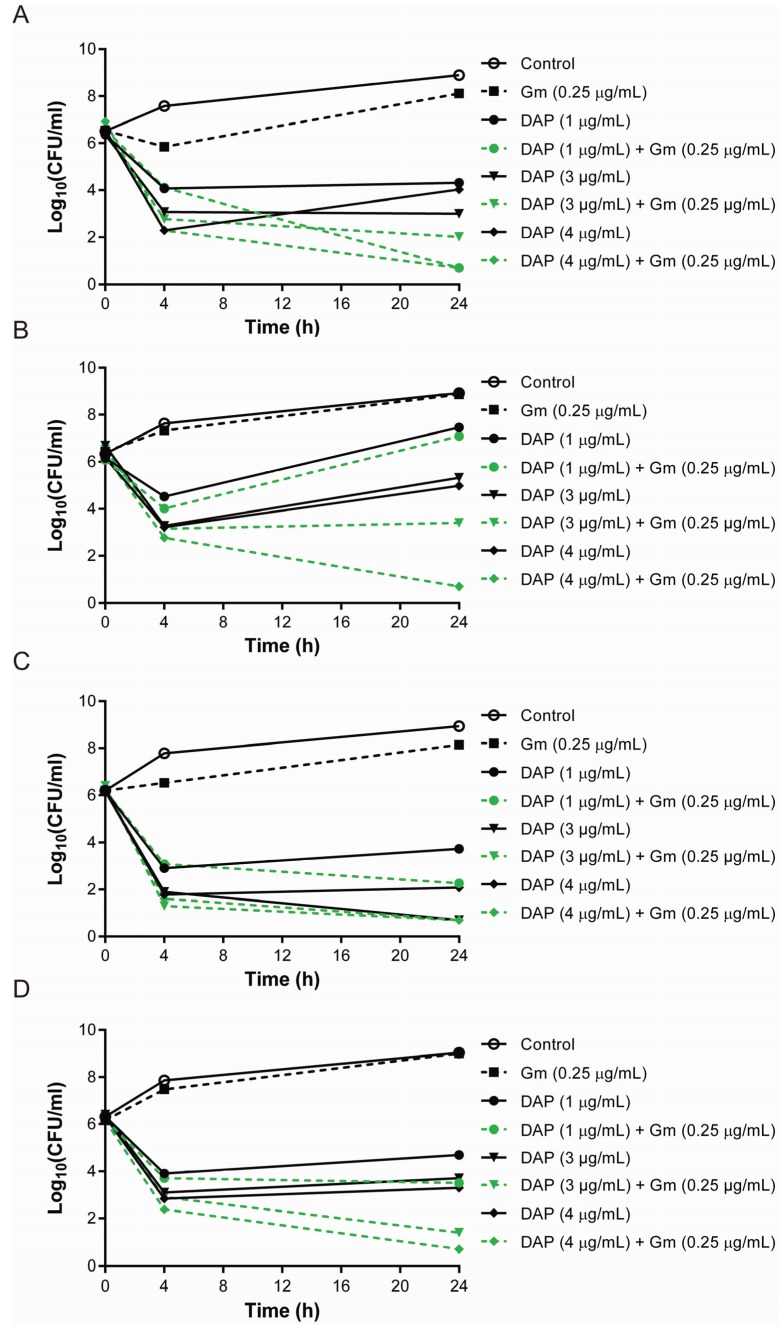
Time-kill studies of four DAP-S, methicillin-resistant *S. aureus* isolates showed enhanced killing with the combination of daptomycin and gentamicin at daptomycin concentrations between 1 μg/mL and 4 μg/mL. (**A**) A6224; (**B**) A9719; (**C**) A8819; and (**D**) A8796.

Lower daptomycin levels are expected in tissue compartments other than blood due to high levels of protein binding of daptomycin and low penetration ratio into tissue compartments [27,28,29,37,38]. The fact that the majority of daptomycin treatment failures described thus far have been due to infections in these non-blood compartments indicates the clinical relevance of these experiments [2]. Of the four DAP-S isolates, three showed enhanced killing between daptomycin and gentamicin (A6224, A9719, A8796) (Figure 1). Enhanced killing was most common at a daptomycin concentration of 4 μg/mL but was observed down to 1 μg/mL. Daptomycin concentration above 4 μg/mL, alone or combined with gentamicin, resulted in rapid bacterial killing whilst significant regrowth was observed for daptomycin concentrations below 1 μg/mL alone and the combination [39].

To assess whether the mechanism of enhanced killing was by the prevention of secondary daptomycin resistance, the MIC of daptomycin was tested on the organisms that had regrowth at 24 h. The MIC of daptomycin increased from 0.25 μg/mL to 1.0 μg/mL for A6224, and from 0.25 μg/mL to 0.5 μg/mL for the other three isolates (A9719, A8819, and A8796), suggesting that combination therapy effectively prevented the emergence of DAP-R.

### 3.2. The Combination of Daptomycin and Gentamicin Effectively Eradicated Daptomycin-Exposed or Vancomycin-Exposed DAP-R S. aureus Isolates

**Figure 2:**
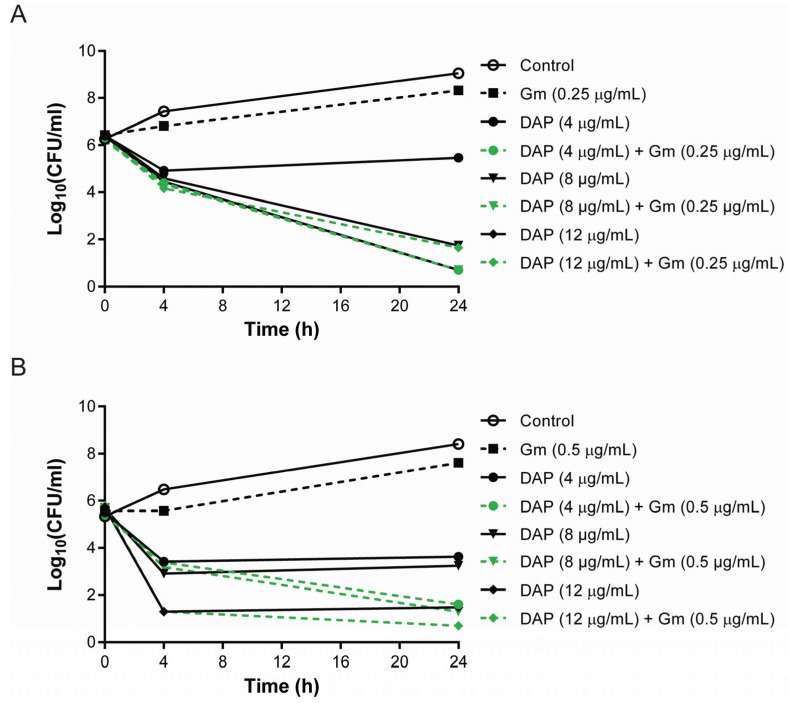
Time-kill studies of two DAP-R *S. aureus* isolates that developed after exposure to daptomycin; (**A**) A8817 and (**B**) A9744.

Limited data are available examining the efficacy of combining daptomycin and gentamicin to kill DAP-R isolates. The genetic mutations associated with the DAP-R strains used in this study are shown in Table S2. The daptomycin-exposed DAP-R isolates (A8817 and A9744) contain SNPs in genes related to phospholipid biogenesis, including *mprF* and cardiolipin synthase 2 (*cls2*) [7]. Although electrorepulsion to DAP caused by alteration of membrane phospholipids was proposed as the mechanism behind DAP-R, more studies are required to elucidate how the alteration of membrane phospholipids leads to DAP-R [7]. Importantly, enhanced killing with the combination of daptomycin 4 μg/mL and gentamicin was seen for the two DAP-R isolates (A8817 and A9744) (Figure 2).

**Figure 3:**
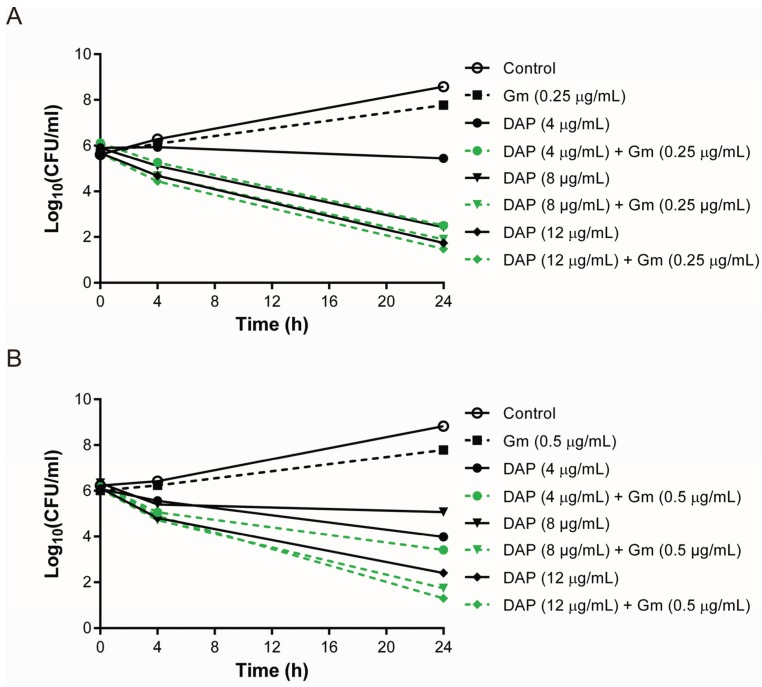
Time-kill studies of two DAP-R *S. aureus* isolates that were exposed to vancomycin only; (**A**) A6226 and (**B**) A9639.

For the vancomycin-exposed DAP-R isolates A6226 and A9639, the synergy between daptomycin and gentamicin was also observed at a daptomycin concentration of 4 μg/mL and 8 μg/mL (Figure 3). Daptomycin concentrations above 8 μg/mL led to substantial killing by daptomycin alone and with the combination. A6226 contains a mutation in *yycI*, which is predicted to be the regulatory protein for the *walKR* operon, and a SNP in *dltA*, which is in the *dlt* operon responsible for D-analylation of wall teichoic acids [10]. Mutations in *walK* and *walR* were shown to be associated with DAP-R and overexpression of the *dlt* operon was found in a daptomycin-exposed, DAP-R isolate, indicating that modification of the cell wall may impact daptomycin susceptibility [11,40]. A9639 has a SNP in *vraG*, which encodes an ABC transporter permease, and a frameshift mutation in *rpsU*, which encodes ribosomal protein S21 [10]. VraG is involved in resistance to host cationic antimicrobial peptides and up-regulation of *vraG* was found in a daptomycin-exposed, DAP-R isolate [41,42]. The integration of a transposon into *rpsU* was recently shown to confer DAP-R in *S. aureus* [43]. Although the genetic mechanisms associated with these DAP-R isolates were diverse, enhanced killing with daptomycin combined with gentamicin was maintained across a range of phenotypes, including DAP-R isolates that had emerged after either vancomycin (VISA phenotype) or daptomycin exposure.

### 3.3. Lower Bactericidal Rates of Daptomycin and Gentamicin against Vancomycin-Exposed DAP-R Isolates Compared to the Rates against Daptomycin-Exposed DAP-R Isolates

We noticed that the degree of bacterial killing at 4 h was significantly lower for DAP-R isolates that had been exposed to vancomycin (VISA isolates A6226 and A9639) compared with DAP-R isolates exposed to daptomycin (A8817 and A9744), with a mean reduction in CFU/mL from baseline of 0.91 CFU/mL and 2.08 CFU/mL, respectively (*p* = 0.049, Kendall’s rank correlation coefficient, STATA, version 7.0, College Station, TX, USA). This may relate to changes in the cell wall with VISA isolates interfering with daptomycin activity, or to cellular changes associated with this phenotype that confer tolerance to antimicrobials [9]. The difference in the rate of bactericidal activity also suggests that the mechanism of DAP-R between these two groups of organisms are likely different.

In contrast to previous studies, we have assessed bactericidal interactions between daptomycin and gentamicin against both DAP-S and DAP-R *S. aureus* isolates using a range of daptomycin concentrations that are likely present in deep-seated infection sites with current dosing [14,15,16,17,18,19,20,21,22,23,24,25,27,28,29,37,38]. We observed, as described by previous investigators, that at higher daptomycin concentrations (>4 μg/mL for susceptible isolates and >8 μg/mL for resistant isolates), rapid killing occurs, with minimal benefit of adding gentamicin [16,24,44]. However, when lower concentrations are used to a point, the addition of gentamicin has significant benefit, often showing equivalent or greater activity than a higher dose of daptomycin alone. Thus, for *S. aureus* bacteremia that is complicated by seeding to other sites, the addition of gentamicin may be of benefit. This needs to be outweighed by the potential nephro- and ototoxicity of aminoglycosides.

Our results also indicate that the addition of gentamicin may be beneficial if daptomycin is used after vancomycin failure to prevent the development of DAP-R. Daptomycin has been approved for the treatment of *S. aureus* bacteremia and right-sided infective endocarditis [1]. To optimize the use of daptomycin in this setting, especially in those with complicated bacteremia who are at greatest risk for therapeutic failure, further studies are required to assess the clinical significance and generalisability of the observed concentration-dependent interaction between daptomycin and gentamicin.

## 4. Conclusions

The combination of gentamicin with daptomycin at concentrations likely present in deep-seated infection sites effectively eradicated *S. aureus* clinical isolates, including DAP-R isolates previously exposed to daptomycin or vancomycin. Combination therapy may provide the synergy required to prevent emergence of resistance in deep-seated, complicated infections, where daptomycin levels are below peak serum concentrations.

## Supplementary Files

Supplementary File 1

## References

[1] Fowler V.G., Boucher H.W., Corey G.R., Abrutyn E., Karchmer A.W., Rupp M.E., Levine D.P., Chambers H.F., Tally F.P., Vigliani G.A. (2006). Daptomycin versus standard therapy for bacteremia and endocarditis caused by staphylococcus aureus. N. Engl. J. Med..

[2] Marty F.M., Yeh W.W., Wennersten C.B., Venkataraman L., Albano E., Alyea E.P., Gold H.S., Baden L.R., Pillai S.K. (2006). Emergence of a clinical daptomycin-resistant staphylococcus aureus isolate during treatment of methicillin-resistant staphylococcus aureus bacteremia and osteomyelitis. J. Clin. Microbiol..

[3] Friedman L., Alder J.D., Silverman J.A. (2006). Genetic changes that correlate with reduced susceptibility to daptomycin in staphylococcus aureus. Antimicrob. Agents Chemother..

[4] Ernst C.M., Staubitz P., Mishra N.N., Yang S.J., Hornig G., Kalbacher H., Bayer A.S., Kraus D., Peschel A. (2009). The bacterial defensin resistance protein mprf consists of separable domains for lipid lysinylation and antimicrobial peptide repulsion. PLoS Pathogens.

[5] Mishra N.N., Yang S.J., Sawa A., Rubio A., Nast C.C., Yeaman M.R., Bayer A.S. (2009). Analysis of cell membrane characteristics of *in vitro*-selected daptomycin-resistant strains of methicillin-resistant staphylococcus aureus. Antimicrob. Agents Chemother..

[6] Rubio A., Moore J., Varoglu M., Conrad M., Chu M., Shaw W., Silverman J.A. (2012). LC-MS/MS characterization of phospholipid content in daptomycin-susceptible and -resistant isolates of staphylococcus aureus with mutations in Mprf. Mol. Membr. Biol..

[7] Peleg A.Y., Miyakis S., Ward D.V., Earl A.M., Rubio A., Cameron D.R., Pillai S., Moellering R.C., Eliopoulos G.M. (2012). Whole genome characterization of the mechanisms of daptomycin resistance in clinical and laboratory derived isolates of staphylococcus aureus. PloS ONE.

[8] Dubrac S., Boneca I.G., Poupel O., Msadek T. (2007). New insights into the walk/walr (YycG/YycF) essential signal transduction pathway reveal a major role in controlling cell wall metabolism and biofilm formation in staphylococcus aureus. J. Bacteriol..

[9] Cui L., Tominaga E., Neoh H.M., Hiramatsu K. (2006). Correlation between reduced daptomycin susceptibility and vancomycin resistance in vancomycin-intermediate staphylococcus aureus. Antimicrob. Agents Chemother..

[10] Cameron D.R., Ward D.V., Kostoulias X., Howden B.P., Moellering R.C., Eliopoulos G.M., Peleg A.Y. (2012). Serine/threonine phosphatase stp1 contributes to reduced susceptibility to vancomycin and virulence in staphylococcus aureus. J. Infectious Dis..

[11] Howden B.P., McEvoy C.R., Allen D.L., Chua K., Gao W., Harrison P.F., Bell J., Coombs G., Bennett-Wood V., Porter J.L. (2011). Evolution of multidrug resistance during staphylococcus aureus infection involves mutation of the essential two component regulator walkr. PLoS Pathogens.

[12] Debbia E., Pesce A., Schito G.C. (1988). *In vitro* activity of LY146032 alone and in combination with other antibiotics against gram-positive bacteria. Antimicrob. Agents Chemother..

[13] Agence francaise de securite sanitaire des produits de s. (2012). Update on good use of injectable aminoglycosides, gentamycin, tobramycin, netilmycin, amikacin. Pharmacological properties, indications, dosage, and mode of administration, treatment monitoring. Med. Mal. Infectieuses.

[14] Snydman D.R., McDermott L.A., Jacobus N.V. (2005). Evaluation of *in vitro* interaction of daptomycin with gentamicin or beta-lactam antibiotics against staphylococcus aureus and enterococci by FIC index and timed-kill curves. J. Chemother..

[15] Tsuji B.T., Rybak M.J. (2006). Etest synergy testing of clinical isolates of staphylococcus aureus demonstrating heterogeneous resistance to vancomycin. Diagn. Microbiol. Infectious Dis..

[16] Tsuji B.T., Rybak M.J. (2005). Short-course gentamicin in combination with daptomycin or vancomycin against staphylococcus aureus in an *in vitro* pharmacodynamic model with simulated endocardial vegetations. Antimicrob. Agents Chemother..

[17] Credito K., Lin G., Appelbaum P.C. (2007). Activity of daptomycin alone and in combination with rifampin and gentamicin against staphylococcus aureus assessed by time-kill methodology. Antimicrob. Agents Chemother..

[18] Baltch A.L., Ritz W.J., Bopp L.H., Michelsen P.B., Smith R.P. (2007). Antimicrobial activities of daptomycin, vancomycin, and oxacillin in human monocytes and of daptomycin in combination with gentamicin and/or rifampin in human monocytes and in broth against staphylococcus aureus. Antimicrob. Agents Chemother..

[19] Baltch A.L., Ritz W.J., Bopp L.H., Michelsen P., Smith R.P. (2008). Activities of daptomycin and comparative antimicrobials, singly and in combination, against extracellular and intracellular staphylococcus aureus and its stable small-colony variant in human monocyte-derived macrophages and in broth. Antimicrob. Agents Chemother..

[20] Miro J.M., Garcia-de-la-Maria C., Armero Y., Soy D., Moreno A., del Rio A., Almela M., Sarasa M., Mestres C.A., Gatell J.M. (2009). Addition of gentamicin or rifampin does not enhance the effectiveness of daptomycin in treatment of experimental endocarditis due to methicillin-resistant staphylococcus aureus. Antimicrob. Agents Chemother..

[21] Entenza J.M., Giddey M., Vouillamoz J., Moreillon P. (2010). *In vitro* prevention of the emergence of daptomycin resistance in staphylococcus aureus and enterococci following combination with amoxicillin/clavulanic acid or ampicillin. Int. J. Antimicrob. Agents.

[22] LaPlante K.L., Woodmansee S. (2009). Activities of daptomycin and vancomycin alone and in combination with rifampin and gentamicin against biofilm-forming methicillin-resistant staphylococcus aureus isolates in an experimental model of endocarditis. Antimicrob. Agents Chemother..

[23] LaPlante K.L., Rybak M.J. (2004). Impact of high-inoculum staphylococcus aureus on the activities of nafcillin, vancomycin, linezolid, and daptomycin, alone and in combination with gentamicin, in an *in vitro* pharmacodynamic model. Antimicrob. Agents Chemother..

[24] DeRyke C.A., Sutherland C., Zhang B., Nicolau D.P., Kuti J.L. (2006). Serum bactericidal activities of high-dose daptomycin with and without coadministration of gentamicin against isolates of staphylococcus aureus and enterococcus species. Antimicrob. Agents chemother..

[25] Rose W.E., Leonard S.N., Rybak M.J. (2008). Evaluation of daptomycin pharmacodynamics and resistance at various dosage regimens against staphylococcus aureus isolates with reduced susceptibilities to daptomycin in an *in vitro* pharmacodynamic model with simulated endocardial vegetations. Antimicrob. Agents Chemother..

[26] Steenbergen J.N., Mohr J.F., Thorne G.M. (2009). Effects of daptomycin in combination with other antimicrobial agents: A review of *in vitro* and animal model studies. J. Antimicrob. Chemother..

[27] Dvorchik B.H., Brazier D., DeBruin M.F., Arbeit R.D. (2003). Daptomycin pharmacokinetics and safety following administration of escalating doses once daily to healthy subjects. Antimicrob. Agents Chemother..

[28] Montange D., Berthier F., Leclerc G., Serre A., Jeunet L., Berard M., Muret P., Vettoretti L., Leroy J., Hoen B. (2014). Penetration of daptomycin into bone and synovial fluid in joint replacement. Antimicrob. Agents Chemother..

[29] Traunmuller F., Schintler M.V., Metzler J., Spendel S., Mauric O., Popovic M., Konz K.H., Scharnagl E., Joukhadar C. (2010). Soft tissue and bone penetration abilities of daptomycin in diabetic patients with bacterial foot infections. J. Antimicrob. Chemother..

[30] Peleg A.Y., Monga D., Pillai S., Mylonakis E., Moellering R.C., Eliopoulos G.M. (2009). Reduced susceptibility to vancomycin influences pathogenicity in staphylococcus aureus infection. J. Infectious Dis..

[31] National Committee for Clinical Laboratory Standards (2000). Methods for Dilution Antimicrobial Susceptibility Testing for Bacteria that Grow Aerobically. Approved Standard M7–A5.

[32] Pillai S.K., Moellering R.C., Eliopoulos G.M., Lorian V. (2005). Antimicrobial Combinations. Antibiotics in Laboratory Medicine.

[33] Zammit M.C., Fiorentino L., Cassar K., Azzopardi L.M., LaFerla G. (2011). Factors affecting gentamicin penetration in lower extremity ischemic tissues with ulcers. Int. J. Lower Extremity Wounds.

[34] Lorentzen H., Kallehave F., Kolmos H.J., Knigge U., Bulow J., Gottrup F. (1996). Gentamicin concentrations in human subcutaneous tissue. Antimicrob. Agents Chemother..

[35] Gill M.A., Cohen J.L., Chenella F.C., Hisayasu G.H., Chandrasoma P., Warnecke G.M., Chung H., Heseltine P.N., Yellin A.E., Berne T.V. (1984). Gentamicin penetration into diseased appendix tissue. Ther. Drug Monit..

[36] Beraud G., le Moal G., Elsendoorn A., Tattevin P., Godet C., Alfandari S., Couet W., Roblot P., Roblot F. (2012). A Survey on the use of gentamicin in infective endocarditis. Eur. J. Clin. Microbiol. Infectious Dis..

[37] Kim A., Suecof L.A., Sutherland C.A., Gao L., Kuti J.L., Nicolau D.P. (2008). *In vivo* microdialysis study of the penetration of daptomycin into soft tissues in diabetic versus healthy volunteers. Antimicrob. Agents Chemother..

[38] Gika H.G., Michopoulos F., Divanis D., Metalidis S., Nikolaidis P., Theodoridis G.A. (2010). Daptomycin determination by liquid chromatography-mass spectrometry in peritoneal fluid, blood plasma, and urine of clinical patients receiving peritoneal dialysis treatment. Anal. Bioanal. Chem..

[39] Jiang J.-H., Peleg A.Y. (2015).

[40] Yang S.J., Kreiswirth B.N., Sakoulas G., Yeaman M.R., Xiong Y.Q., Sawa A., Bayer A.S. (2009). Enhanced expression of dltabcd is associated with the development of daptomycin nonsusceptibility in a clinical endocarditis isolate of staphylococcus aureus. J. Infectious Dis..

[41] Yang S.J., Bayer A.S., Mishra N.N., Meehl M., Ledala N., Yeaman M.R., Xiong Y.Q., Cheung A.L. (2012). The staphylococcus aureus two-component regulatory system, grars, senses and confers resistance to selected cationic antimicrobial peptides. Infection Immunity.

[42] Camargo I.L., Neoh H.M., Cui L., Hiramatsu K. (2008). Serial daptomycin selection generates daptomycin-nonsusceptible staphylococcus aureus strains with a heterogeneous vancomycin-intermediate phenotype. Antimicrob. Agents Chemother..

[43] Blake K.L., O’Neill A.J. (2013). Transposon library screening for identification of genetic loci participating in intrinsic susceptibility and acquired resistance to antistaphylococcal agents. J. Antimicrob. Chemother..

[44] Akins R.L., Rybak M.J. (2000). *In vitro* activities of daptomycin, arbekacin, vancomycin, and gentamicin alone and/or in combination against glycopeptide intermediate-resistant staphylococcus aureus in an infection model. Antimicrob. Agents Chemother..

