# Supplementary material for: Daptomycin-Nonsusceptible Staphylococcus aureus: The Role of Combination Therapy with Daptomycin and Gentamicin
Source: Genes (Basel). 2015 Nov 30;6(4):1256–67. doi: 10.3390/genes6041256 (PMC4690039; doi:10.3390/genes6041256)
Supplement: Supplementary File 1 [file genes-06-01256-s001.pdf]

Supplementary Materials

Table S1. Studies related to daptomycin and gentamicin combination therapy.

Author (Year)	Isolates Tested	Methods	Dose	Findings
		in vitro combination studies		
Snydman *et al.*, (2005) [1]	20 MSSA (DAP-S)<br>20 MRSA (DAP-S)	Checkerboard (fractional inhibitory concentration index)	DAP 0.01–8 µg/mL (0.0625–4× MIC)<br>Gen 0.01–4096 µg/mL (0.0625–4× MIC)	DAP + Gen synergy observed for 1 MRSA strain. No synergistic inhibition of bacterial growth was observed among other isolates.
Tsuji *et al.*, (2006) [2]	1 GISA (DAP-S)<br>1 hGISA (DAP-S)	Time-kill<br>E-test	DAP 0.25 µg/mL (0.5× MIC)<br>Gen 1 µg/mL (0.5× MIC)	DAP + Gen and DAP alone were indifferent in Time-kill assays but showed additive effects using E-test
Credito *et al.*, (2007) [3]	8 MSSA (DAP-S)<br>24 MRSA (DAP-S)<br>3 VISA (DAP-R)<br>3 VRSA (DAP-S)	Time-kill	DAP 0.125–0.5 µg/mL for isolates with DAP-S (0.25–0.5× MIC)<br>DAP 0.5–4 µg/mL for isolates with DAP-R (0.25–0.5× MIC)	DAP + Gen showed synergy in killing 4 MSSA, 17 MRSA, 2VISA and 2 VRSA. Additive effects were observed among other isolates.
Baltch *et al.*, (2007) [4]	1 MRSA (DAP-S)	Extracellular and Intracellular killing of staphylococci in human monocytes culture	DAP 0.5 µg/mL (1× MIC)<br>Gen 1µg/mL (0.5× MIC)	DAP + Gen showed synergy for killing staphylococci inside human monocytes and staphylococci in broth.
Baltch *et al.*, (2008) [5]	2 Pairs of SCV and parental isolates (All DAP-S)	Extracellular and Intracellular killing of staphylococci in human monocytes culture	DAP 0.5–1 µg/mL (1× MIC)<br>Gen 1–8 µg/mL (0.5× MIC)	DAP + Gen showed synergy for killing intracellular parental isolates and 1 SCV. For extracellular killing, DAP alone efficiently killed 1 SCV and the parental isolates.
Miro *et al.*, (2009) [6]	3 MRSA (DAP-S)	Time-kill	DAP 0.25–1 µg/mL (0.5–2× MIC)<br>Gen 0.5–2 µg/mL	DAP + Gen showed synergy against all strains compared to monotherapy.
Entenza *et al.*, (2010) [7]	4 MRSA (DAP-S)<br>2 GISA (1 DAP-S, 1 DAP-R)	Progressive DAP resistance selection followed by MIC test	2-fold stepwise increasing DAP<br>Gen 0.0625–32µg/mL (0.25× MIC)	Addition of Gen did not prevent DAP-R among all the tested isolates.

Table S1. *Cont.*

**Author (Year)**	**Isolates Tested**	**Methods**	**Dose**	**Findings**
		in vitro pharmacodynamic models		
LaPlante *et al.*, (2004) [8]	1 MSSA (DAP-S)<br>1 MRSA (DAP-S)	Simulated endocardial vegetation	DAP 6 mg/kg/day<br>Gen 1.3 mg/kg/day	DAP + Gen increased bactericidal rates in the first 24 h and eradicated staphylococci as efficient as DAP monotherapy after 72 h
Tsuji *et al.*, (2006) [2]	1 MSSA (DAP-S)<br>1 MRSA (DAP-S)	Simulated endocardial vegetation	DAP 6 or 8 mg/kg<br>3 dose of Gen 1 mg/kg or 1 dose of Gen 5 mg/kg	Gen enhanced bactericidal activity of DAP in all combinations.
DeRyke *et al.*, (2006) [9]	1 MRSA (DAP-S)<br>1 CA-MRSA (DAP-S)<br>1 MSSA (DAP-S)	Serum bactericidal titers (Serum from volunteers with DAP alone or DAP + Gen)	DAP 6 mg/kg<br>Gen 1 mg/kg	Addition of Gen did not enhance serum bactericidal titers against staphylococci compared to DAP monotherapy.
Rose *et al.*, (2008) [10]	3 DAP-S<br>4 DAP-R	Simulated endocardial vegetation	DAP 6 mg/kg/day (DAP6)<br>DAP 10 mg/kg/day (DAP10)<br>Gen 5 mg/kg/day	2 DAP-S isolates were eradicated by DAP6 alone or DAP6 + Gen. 1 DAP-S isolate was only eradicated by DAP6 + Gen.<br>For DAP-R isolates, only 1 isolate was eradicated by DAP6 + Gen. 2 isolates were eradicated by DAP10 alone or DAP10 + Gen.
LaPlante *et al.*, (2009) [11]	2 MRSA (DAP-S)	Simulated endocardial vegetation	DAP 6 mg/kg/day<br>Gen 1.3 mg/kg/day	DAP monotherapy has better *in vitro* activity than DAP + Gen within the first 24 h.
		Animal model		
Miró *et al.*, (2009) [6]	1 MRSA (DAP-S)	Rabbit infective endocarditis	DAP 6 mg/kg<br>Gen 1 mg/kg	DAP + Gen eradicated staphylococci as efficient as DAP monotherapy.

DAP: daptomycin; Gen: gentamicin; DAP-S: daptomycin-susceptible; DAP-R: daptomycin-resistant; MSSA: methicillin-susceptible *Staphylococcus aureus*; MRSA: methicillin-resistant *Staphylococcus aureus*; MIC: minimum inhibitory concentration; GISA: glycopeptides-intermediate *Staphylococcus aureus*; hGISA: heterogeneous glycopeptides-intermediate *Staphylococcus aureus*; VISA: vancomycin-intermediate *Staphylococcus aureus*; VRSA: vancomycin-resistant *Staphylococcus aureus*; SCV: small-colony variant.

Table S2. Mutations identified between isogenic *S. aureus* strain pairs used in this study [12,13].

Gene	Protein	Function	[a] Mutation		Reference
			Type	Change	
1. A6224/A6226					
dltA	D-alanine-poly(phosphoribitol) ligase, subunit 1	Cell wall/outer membrane metabolism	[b] SNP	Ser38Arg	[13]
yycI	Regulatory protein, WalKR operon	Cell wall/outer membrane metabolism	Deletion	Frameshift	
atl	Bifunctional autolysin	Cell wall/outer membrane metabolism	SNP	Ser752Ser	
arlS	Histidine-kinase	Signal transduction	Insertion	Frameshift	
mraW	S-adenosyl-methyltransferase	Amino acid metabolism/transport	Deletion	Frameshift	
pcrA	ATP-dependent helicase	DNA replication and repair	SNP	Glu455Glu	
tilS	tRNA(ile)-lysidine synthetase	Nucleic acid metabolism/transport	SNP	Met128Ile	
SA0587	PsaA adhesion homologue	Inorganic ion transport/metabolism	Deletion	Frameshift	
SA1528	Universal stress protein	Stress response	SNP	Leu105Leu	
SA0837	2-siopropylmalate synthase	Amino acid metabolism/transport	Insertion	Frameshift	
SA1778	Hypothetical protein	Unknown	Deletion	Frameshift	
SA0349	Hypothetical protein	Unknown	SNP	Pro474Ser	
SA0668	Hypothetical protein	Unknown	SNP	Val41Ile	
2. A9719/A9744					
mprF	Lysylphosphatidylglycerol synthetase	Oxacillin resistance related protein	SNP	Ser337Leu	[12]
cls2	Cardiolipin synthetase	Phosphatidylcholine-hydrolysing phospholipase D	SNP	Ala23Val	
atl	N-acetylmuramoyl-L-alanine amidase	Bi-funtional autolysin involved in cell envelope biogenesis	Insertion	Frameshift	
agrC	Accessory gene regulator C	Sensor histidine kinase	Insertion	Frameshift	
stp1	Protein phosphatase 2C domain-containing protein	Serine/threonine specific protein phosphatase	SNP	Met99Ile	
SA1264	Conserved hypothetical protein	Unknown function	SNP	Gly47Glu	
3. A8819/A8817					
mprF	Lysylphosphatidylglycerol synthetase	Oxacillin resistance related protein	SNP	Thr345Ile	[12]
cls2	Cardiolipin synthetase	Phosphatidylcholine-hydrolysing phospholipase	SNP	Phe60Ser	
leuS	Leucyl-tRNA synthetase	Incorperation of leucine into tRNA	SNP	Asn468Asp	
mnaA	UDP-N-acetylglucosamine 2-epimerase	Sialic acid synthesis	SNP	Arg302Ser	
ispA	Geranyltranstransferase	Polyketide biosynthesis	Deletion	Frameshift	

Table S2. *Cont.*

Gene	Protein	Function	[a] Mutation		Reference
			Type	Change	
		4. A9635/A9639			
vraG	ABC transporter permease	ABC transporter, defense	SNP	Ala580Val	[13]
rsbW	Ser-protein kinase, anti-σ β factor	Transcriptional regulation	Deletion	88Ser/89Phe	
rpsU	Ribosomal protein S21	Translation	Deletion	Frameshift	
ilvC	Ketol-acid reductoisomerase	Amino acid metabolism/transport	Deletion	30Gln/31Gly	

[a] Nucleotide and amino acid mutations are those identified in the daptomycin-exposed strains compared to their isogenic parent strains; [b] SNP—Single nucleotide polymorphism.

References

1. Snydman, D.R.; McDermott, L.A.; Jacobus, N.V. Evaluation of *in vitro* interaction of daptomycin with gentamicin or beta-lactam antibiotics against staphylococcus aureus and enterococci by fic index and timed-kill curves. *J. Chemother.* **2005**, *17*, 614–621.
2. Tsuji, B.T.; Rybak, M.J. Etest synergy testing of clinical isolates of staphylococcus aureus demonstrating heterogeneous resistance to vancomycin. *Diagn. Microbiol. Infect. Dis.* **2006**, *54*, 73–77.
3. Credito, K.; Lin, G.; Appelbaum, P.C. Activity of daptomycin alone and in combination with rifampin and gentamicin against staphylococcus aureus assessed by time-kill methodology. *Antimicrob. Agents Chemother.* **2007**, *51*, 1504–1507.
4. Baltch, A.L.; Ritz, W.J.; Bopp, L.H.; Michelsen, P.B.; Smith, R.P. Antimicrobial activities of daptomycin, vancomycin, and oxacillin in human monocytes and of daptomycin in combination with gentamicin and/or rifampin in human monocytes and in broth against staphylococcus aureus. *Antimicrob. Agents Chemother.* **2007**, *51*, 1559–1562.
5. Baltch, A.L.; Ritz, W.J.; Bopp, L.H.; Michelsen, P.; Smith, R.P. Activities of daptomycin and comparative antimicrobials, singly and in combination, against extracellular and intracellular staphylococcus aureus and its stable small-colony variant in human monocyte-derived macrophages and in broth. *Antimicrob. Agents Chemother.* **2008**, *52*, 1829–1833.
6. Miro, J.M.; Garcia-de-la-Maria, C.; Armero, Y.; Soy, D.; Moreno, A.; del Rio, A.; Almela, M.; Sarasa, M.; Mestres, C.A.; Gatell, J.M.; *et al.* Addition of gentamicin or rifampin does not enhance the effectiveness of daptomycin in treatment of experimental endocarditis due to methicillin-resistant staphylococcus aureus. *Antimicrob. Agents Chemother.* **2009**, *53*, 4172–4177.

7. Entenza, J.M.; Giddey, M.; Vouillamoz, J.; Moreillon, P. *In vitro* prevention of the emergence of daptomycin resistance in staphylococcus aureus and enterococci following combination with amoxicillin/clavulanic acid or ampicillin. *Int. J. Antimicrob. Agents* **2010**, *35*, 451–456.
8. LaPlante, K.L.; Rybak, M.J. Impact of high-inoculum staphylococcus aureus on the activities of nafcillin, vancomycin, linezolid, and daptomycin, alone and in combination with gentamicin, in an *in vitro* pharmacodynamic model. *Antimicrob. Agents Chemother.* **2004**, *48*, 4665–4672.
9. DeRyke, C.A.; Sutherland, C.; Zhang, B.; Nicolau, D.P.; Kuti, J.L. Serum bactericidal activities of high-dose daptomycin with and without coadministration of gentamicin against isolates of staphylococcus aureus and enterococcus species. *Antimicrob. Agents Chemother.* **2006**, *50*, 3529–3534.
10. Rose, W.E.; Leonard, S.N.; Rybak, M.J. Evaluation of daptomycin pharmacodynamics and resistance at various dosage regimens against staphylococcus aureus isolates with reduced susceptibilities to daptomycin in an *in vitro* pharmacodynamic model with simulated endocardial vegetations. *Antimicrob. Agents Chemother.* **2008**, *52*, 3061–3067.
11. LaPlante, K.L.; Woodmansee, S. Activities of daptomycin and vancomycin alone and in combination with rifampin and gentamicin against biofilm-forming methicillin-resistant staphylococcus aureus isolates in an experimental model of endocarditis. *Antimicrob. Agents Chemother.* **2009**, *53*, 3880–3886.
12. Peleg, A.Y.; Miyakis, S.; Ward, D.V.; Earl, A.M.; Rubio, A.; Cameron, D.R.; Pillai, S.; Moellering, R.C., Jr.; Eliopoulos, G.M. Whole genome characterization of the mechanisms of daptomycin resistance in clinical and laboratory derived isolates of staphylococcus aureus. *PLoS ONE* **2012**, *7*, e28316.
13. Cameron, D.R.; Ward, D.V.; Kostoulias, X.; Howden, B.P.; Moellering, R.C., Jr.; Eliopoulos, G.M.; Peleg, A.Y. Serine/threonine phosphatase stp1 contributes to reduced susceptibility to vancomycin and virulence in staphylococcus aureus. *J. Infect. Dis.* **2012**, *205*, 1677–1687.

© 2015 by the authors; licensee MDPI, Basel, Switzerland. This article is an open access article distributed under the terms and conditions of the Creative Commons Attribution license (http://creativecommons.org/licenses/by/4.0/).
